# A systematic review of the effects of low-frequency repetitive transcranial magnetic stimulation on cognition

**DOI:** 10.1007/s00702-016-1592-8

**Published:** 2016-08-08

**Authors:** Claudia Lage, Katherine Wiles, Sukhwinder S. Shergill, Derek K. Tracy

**Affiliations:** 1 Cognition, Schizophrenia and Imaging Laboratory, The Institute of Psychiatry, Psychology and Neuroscience, King’s College London, London, UK; 2 King’s College London School of Medicine, London, UK; 3 South London and Maudsley NHS Foundation Trust, London, UK; 4 Oxleas NHS Foundation Trust, London, UK

**Keywords:** rTMS, Cognition, Systematic review, Neuropsychiatry

## Abstract

rTMS is increasingly used for a variety of neuropsychiatric conditions. There are data to support ‘fast’ rTMS (≥10 Hz) having some positive effects on cognitive functioning, but a dearth of research looking at any such effects of ‘slow’ rTMS. This question is important as cognitive dysfunction accompanies many neuropsychiatric conditions and neuromodulation that potentially enhances or hinders such functioning has important clinical consequences. To determine cognitive effects of slow (≤1 Hz) rTMS, a systematic review of randomized control trials assayed cognition in neurological, psychiatric, and healthy volunteer ≤1 Hz rTMS paradigms. Both active (fast rTMS) and placebo comparators were included. 497 Records were initially obtained; 20 met inclusion criteria for evaluation. Four major categories emerged: mood disorders; psychotic disorders; cerebrovascular accidents; and ‘other’ (PTSD, OCD, epilepsy, anxiety, and tinnitus). Cognitive effects were measured across several domains: attention, executive functioning, learning, and psychomotor speed. Variability of study paradigms and reporting precluded meta-analytical analysis. No statistically significant improvement or deterioration was consistently found in any cognitive domain or illness category. These data support the overall safety of rTMS in not adversely affecting cognitive functioning. There are some data indicating that rTMS might have cognitive enhancing potential, but these are too limited at this time to make any firm conclusions, and the literature is marked by considerable heterogeneity in study parameters that hinder interpretation. Greater consensus is required in future studies in cognitive markers, and particularly in reporting of protocols. Future work should evaluate the effects of rTMS on cognitive training.

## Introduction

Transcranial magnetic stimulation (TMS) is a non-invasive cortical modulating tool, where a fluctuating magnetic field induces an electrical current that depolarises underlying neurons (Wassermann et al. 2008). Repetitive TMS (rTMS) can be applied as either low (≤1 Hz) or high (≥5 Hz) frequency; the former considered typically inhibitory to underlying neurons, the latter excitatory (Pell et al. 2011). The effects on distal but functionally connected regions may be more complex (Tracy et al. 2011, Tracy et al. 2014).

rTMS alters synaptic plasticity through long-term potentiation (LTP) and long-term depression (LTD) changes (Hoogendam et al. 2010); however, the underlying mechanisms of these effects are not fully understood (Pell et al. 2011; Ridding and Rothwell 2007). Rodent studies demonstrate that rTMS increases the expression of genes important for synaptic plasticity, such as c-Fos (Aydin-Abidin et al. 2008; Doi et al. 2001), but at present, data on rTMS-induced intracellular changes in gene expression, protein synthesis, or other alterations to secondary messenger signalling are largely understudied (Hulme et al. 2013).

The ability to modulate cortical activity—relatively easily, painlessly, and without the use of a general anaesthetic—has garnered significant interest concerning potential clinical application. In psychiatric populations, the utility of rTMS in depression and psychosis has been most studied, and a recent systematic review of meta-analyses supports a modest effectiveness in both of these conditions (Hovington et al. 2013). Nascent positive results have also been obtained in the treatment of anorexia nervosa (Van den Eynde et al. 2013), bulimia nervosa (Van den Eynde et al. 2010), obsessive–compulsive disorder (OCD) (Berlim et al. 2013; Greenberg et al. 1997; Mantovani et al. 2010), tinnitus (Khedr et al. 2010; Kleinjung et al. 2005; Landgrebe et al. 2013; Langguth et al. 2003), and stroke (Khedr et al. 2009; Kim et al. 2006; Takeuchi et al. 2008). However, the literature is overall marked by often conflicting results between trials and considerable methodological concerns about study size and the lack of consensus on optimal rTMS technique parameters (Tracy and David 2015).

In cognitive neuroscience, TMS has been utilised as a tool to disrupt normal cortical activity as a means of better elucidating various cognitive processes (Miniussi and Rossini 2011; Tracy et al. 2015; Wassermann et al. 2008). Typically, non-repetitive TMS is applied during the execution of a cognitive task (so-called “online TMS”), and a transient disruption of normal functioning (a “virtual lesion”) is induced allowing inferences to be made about the role of the stimulated brain area in the cognitive task (Miniussi et al. 2010; Wassermann et al. 2008). For example, Gough et al. 2005 determined that three pulses of TMS to the anterior left inferior frontal cortex (LIFC) delivered at 100 ms intervals caused a significant slowing of response in a semantic judgement task, but not in a phonological judgment task; and conversely that TMS given to the posterior LIFC caused a significant slowing of response in the phonological task, but not in the semantic one (Gough et al. 2005). The effects of offline stimulation on cognitive functioning, with task execution and TMS stimulation temporally dissociated, have also been investigated (Demirtas-Tatlidede et al. 2013; Miniussi and Rossini 2011). Studies have largely focussed on cognitive recovery after stroke, prolonged psychiatric disease, or traumatic brain injury. No conclusive evidence is currently available regarding the use of offline non-invasive brain stimulation for the rehabilitation of such neuropsychiatric disease, though undoubtedly such work is still at a nascent stage (Demirtas-Tatlidede et al. 2013).

Most data on cognitive effects of TMS in studies on participants with mental illness come from clinical trials where they are often reported as part of safety and side-effects assessments (Demirtas-Tatlidede et al. 2013). Contrary to electroconvulsive therapy (ECT) (Schulze-Rauschenbach et al. 2005), the majority of studies show that rTMS has no clear deleterious effects, though the secondary nature of such data collection means that overall there is a dearth of information on this topic (Anderson et al. 2006; Guse et al. 2010). Some clinical trials have found rTMS to be associated with improvements across several cognitive domains (Fitzgerald et al. 2009; Hoppner et al. 2003). For example, Mogg et al. (2007) found that 10 Hz rTMS led to a significant improvement in verbal learning among patients with schizophrenia, whilst Martis et al. (2003) found that 10 Hz rTMS resulted in significant improvements across various cognitive domains, including executive functioning and memory among patients with depression (Mogg et al. 2007).

In addition to focusing on psychiatric applications, an increasing number of studies are now addressing the potential therapeutic effects of rTMS in the context of cognitive neurorehabilitation (Miniussi and Rossini 2011; Stuss 2011). Indeed, 10 Hz rTMS was associated with a significant improvement in executive functioning among patients with cerebrovascular disease (Rektorova et al. 2005). Problematically, depression, schizophrenia, and cerebrovascular disease are associated with illness-driven state-based cognitive difficulties, for example, driven through neuropsychological processes, such as low mood, impaired attention, and concentration. Thus, rTMS-induced improvements in cognition may be—at least partially—through ameliorating individuals’ mental states rather than primarily enhancing cognition.

## Aim

To date, the majority of studies investigating the effects of rTMS on cognitive functioning have used high-frequency stimulation, though this might be an artefact of fast rTMS being the most common paradigm, particularly in depression. A systematic review found that, in most studies, high-frequency rTMS had no significant effect on cognition (Guse et al. 2010). There was, however, variation: several studies reported improvements and three studies deterioration in cognitive functioning. Further studies have demonstrated no effect of high-frequency rTMS on working memory (Guse et al. 2013) and verbal and figural fluency (Schaller et al. 2013) in healthy patients. However, Guse et al. (2013) suggest a role for high-frequency rTMS in cognitive neuroprotection from the loss of working memory in schizophrenia. To date, there has been no systematic review of the effects of low-frequency rTMS on cognitive functioning despite low-frequency rTMS remaining a common clinical paradigm, particularly in psychosis. This paper aimed to systematically review the literature for the effects of slow (<1 Hz) rTMS in cognition.

## Method

### Eligibility criteria

These were defined a priori using the PICOS components (participants, interventions, comparators, outcomes, and study design) as defined by the PRISMA statement on systematic reviews (Liberati et al. 2009).

*Participants* Subjects without pervasive developmental disorders and neurodegenerative diseases; with any psychiatric disorder, neurological condition; and healthy were considered for inclusion. No restrictions regarding age or other population characteristics were applied.

*Interventions* Only studies using 1 Hz rTMS, which is utilised by the majority of low-frequency rTMS studies, were considered. No restrictions regarding other rTMS parameters were applied. Studies with online rTMS paradigms designed to induce virtual lesions were excluded, as these typically evaluate very specific neurocognitive subdomains, and their generalisability to cognitive functioning in the wider clinical populations is challengeable; and studies with 1 Hz rTMS administered in combination with other frequencies were also excluded.

*Comparators* Both active (e.g., high-frequency rTMS) and placebo (e.g., sham rTMS) interventions were considered. No restrictions were applied.

*Outcomes* Studies with one or more objective assessments of cognitive functioning were considered. No further restrictions were applied.

*Study design* Only randomized trials were considered for inclusion.

### Literature search

Four separate electronic searches were performed using Medline (Ovid), Embase (Ovid), PsycInfo (Ovid), and the Cochrane Library as databases. Databases were last searched in September 2014. The following search terms were used: repetitive transcranial magnetic stimulation, rTMS, cognition, neurocognitive, neuropsychological, attention, reaction time, executive function, memory, learning, and processing speed. The search limits applied were English language, publication years from 1992 until 2014 and randomized trials.

### Study selection

All records obtained from the electronic searches were sequentially screened on the basis of title and abstract: those that clearly did not meet the eligibility criteria were excluded, and duplicates were removed. The full texts were examined by two of the authors (C.L. and D.K.T.) for the remaining studies.

### Data extraction

A data extraction form was developed based on the guidelines of the Cochrane Collaboration (Green 2011). The form was piloted on half of the included studies and revised accordingly. The following data were extracted from each: source, study design, total number of participants, sex, age, diagnosis, medication, location (of administered rTMS), number of sessions per week, frequency, coil type, total number of pulses per session, train duration and inter-train interval, intensity, comparator/control group, outcomes, and results.

### Risk of bias assessment

The Cochrane Collaboration’s bias assessment software was used to measure the validity of each included study (Green 2011).

### Data analysis

Due to overall heterogeneity of participants, rTMS parameters, comparator groups, and cognitive measurements, statistical combination of results for meta-analytical comparison was not considered valid. A narrative synthesis was deemed the most suitable method of data analysis. The following elements were addressed: design paradigm, neurocognitive effects, and risk of bias. Studies were organised by clinical groups namely: mood disorders, psychotic disorders, and stroke, with a fourth group, including all other single studies (epilepsy, OCD, tinnitus, and healthy participants). Full comparison of individual studies, including study size, design, parameters, and cognitive measures, used are available in the supplementary material.

## Results

### Search and selection of studies

**Fig. 1:**
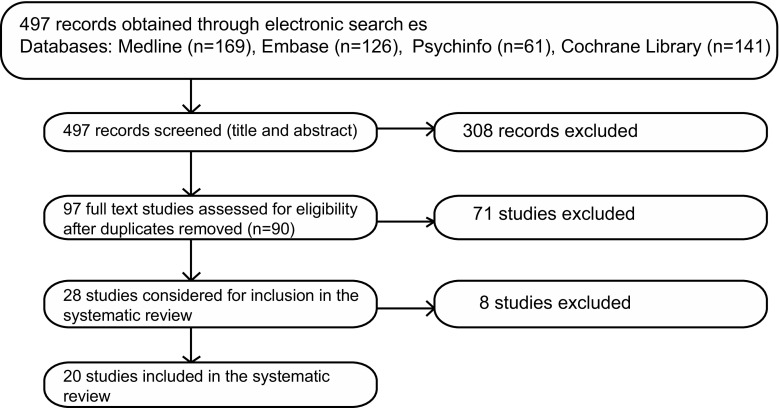
Flow diagram of study selection

497 records were initially obtained, and 308 excluded, because of evident lack of relevance to the eligibility criteria. 90 duplicates were removed, and the eligibility of the remaining 99 studies was further assessed: 41 studies were excluded, because they did not meet the intervention criteria; 2 did not meet comparator criteria; 30 did not meet outcome criteria; 5 did not meet the study design criteria; and 1 study was excluded, because of overlap of patient data with one of the included studies. This initial search yielded 20 studies (Fig. 1).

### Characteristics of included studies

Fifteen out of the 20 included studies were randomized, double-blind, sham-controlled studies, with two of these having a cross-over design. The remaining five studies consisted of two randomized double-blind non-sham-controlled cross-over studies; one randomized blind sham-controlled cross-over study; one randomized blind study; one blind randomized sham-controlled study; and one randomized open study (Table 1). Overall, the studies had small sample sizes, with the lowest number being four participants and the highest 60 (mean 29.95, SD 19.13). The participants’ ages ranged from 16 to 79 (not all studies reported a mean). Eight studies included patients with mood disorders; five included patients with psychotic disorders (schizophrenia and schizoaffective disorder); four studies involved stroke patients; and the remaining were individual studies of epilepsy, OCD, tinnitus, with one comprised healthy participants. The supplementary tables provide details of the extracted data.

**Table 1:** Characteristics of included studies

# of studies	Studies	Randomized	Double-blind	Single-blind	Sham-control	Cross-over	Open
13	Fitzgerald et al. (2005), Fregni et al. (2006a, b), Hoffman et al. (2005, 2013), Hoppner et al. (2003), Januel et al. (2006), Kang et al. (2009), Kim et al. (2010), Koren et al. (2001), Schneider et al. (2008), Thiel et al. (2013), Waldowski et al. (2012)	×	×		×		
2	McIntosh et al. (2004), Speer et al. (2001)	×	×		×	×	
2	Fitzgerald et al. (2009), Little et al. (2000)	×	×			×	
1	Smith et al. (2007)	×		×	×	×	
1	Watts et al. (2012)	×		×	×		
1	Hansen et al. (2011)	×					×

The 1 Hz rTMS parameters differed substantially in the location of the stimulus, the number of rTMS sessions, the number of pulses per session, the motor threshold (MT), and the outcome measures. Areas to which rTMS was applied included the primary motor cortex (PMC), the left temporoparietal cortex (TPC), and the left or right dorsolateral prefrontal cortex (DLPFC).

The latter was the most frequently selected option. The total number of rTMS sessions ranged from one to 20, with ten sessions (five per week) being the mode. The intervention duration additionally varied from 1 day to 4 weeks. In 16 of the 20 included studies, the total number of pulses per session and the train duration/inter-train interval were either not reported or not sufficiently clearly reported. Amongst the remaining studies, these parameters differed substantially. The intensity of rTMS also varied across studies, however, in the majority, it ranged between 80 and 110 % of the MT. Three different comparator groups were used: high-frequency rTMS (10 or 20 Hz), sham stimulation, and, in one study, electroconvulsive therapy (ECT). 40 different tests assessing cognitive domains were used across studies.

### Timing of cognitive measure

The time between rTMS and neurocognitive testing was evaluated. All 20 studies performed baseline testing prior to rTMS intervention. 18 of the 20 included studies performed cognitive testing immediately after completing the last session of rTMS. Of the other two, one (Januel et al. 2006) performed testing half way through the 4 weeks of rTMS; and one (Thiel et al. 2013) did post-treatment testing, but did not clarify when this occurred. 11 out of the 20 papers reported follow-up cognitive assessment after rTMS. The mean time from the final session to follow-up was 32.3 days (SD 29.08). The shortest time to follow-up was 3 days and the longest 105 days (15 weeks). 7 studies did not report follow-up and 2 were not clear as to the follow-up. One of the studies did not report follow-up (Januel et al. 2006); one of the studies was not clear as to follow-up (Thiel et al. 2013).

### Neurocognitive effects of low-frequency rTMS

Due to the variety of outcome measures reported, the data were tabulated according to mental illness, broadly: mood disorders, psychotic disorders, cerebrovascular accident, and “other” (encompassing PTSD, OCD, epilepsy, anxiety, and tinnitus). A further categorization into neurocognitive domains assessed by the outcome measure used in each study was performed to facilitate cross-comparison. These categories were attention, executive function/working memory, learning and memory, and psychomotor speed and processing. No statistically significant improvement or deterioration was found in any one cognitive domain across the disease categories. Two papers (Fitzgerald et al. 2005; Hansen et al. 2011) reported statistically significant deterioration and in the cognitive domain of verbal fluency and retrieval. Furthermore, the majority of papers reported no significant improvement across the cognitive domains (Table 2).

**Table 2:** Cognitive effects of low-frequency rTMS

Disorder	Cognitive domain	Improvement	No effect (ns)	Deterioration
Mood disorder	Attention			
	Selective/focussed attention		Speer et al. (2001)*, Little et al. (2000)**, Januel et al. (2006)*, Hoffman et al. (2005)*	
	Sustained attention/concentration	Hoppner et al. (2003)*	Speer et al. (2001)*, Hansen et al. (2011)***	
	Executive functions/working memory			
	Working memory (short-term storage/manipulation/monitoring)	Fitzgerald et al. (2009)*	Hoffman et al. (2005)*, Watts et al. (2012)*	
	Cognitive flexibility	Fitzgerald et al. (2009)*	Speer et al. (2001)*, Januel et al. (2006)*	
	Verbal fluency/retrieval	Fitzgerald et al. (2009)*	Little et al. (2000)**, (Speer et al. 2001)*	Hansen et al. (2011)***
	Learning and memory (intermediate-/long-term storage)			
	Verbal learning + memory	Little et al. (2000)*	Hoffman et al. (2005)*, Hansen et al. (2011)***	
	Spatial learning + memory/objective learning + memory		Little et al. (2000)**, Speer et al. (2001)*, Januel et al. (2006)*	
	(Visual) associative learning + memory	Hansen et al. (2011)***	Fitzgerald et al. (2009)*	
	Psychomotor speed			
	Psychomotor speed/processing speed	Hoppner et al. (2003)*	Speer et al. (2001)*, Januel et al. (2006)*, Watts et al. (2012)*	
Psychotic illness	Attention			
	Selective/focussed attention		Hoffman et al. (2005)*	
	Executive functions/working memory			
	Working memory (short-term storage/manipulation/monitoring)		Hoffman et al. (2005)*	
	Cognitive flexibility	Hoffman et al. (2013)*, Schneider et al. (2008)*	Hoffman et al. (2005)*	
	Verbal fluency/retrieval			Fitzgerald et al. (2005)*
	Learning and memory (intermediate-/long-term storage)			
	Verbal learning + memory		Hoffman et al. (2013)*, Fitzgerald et al. (2005)*, McIntosh et al. (2004)*	
	Psychomotor speed			
	Psychomotor speed/Processing speed		Hoffman et al. (2005)*	
Stroke	Attention			
	Alertness/simple reaction	Waldowski et al. (2012)***		
	Selective/focussed attention		Fregni et al. (2006a)***, Kim et al. (2010)***	
	Executive functions/working memory			
	Working memory (short-term storage/manipulation/monitoring)		Fregni et al. (2006a)***, Kim et al. (2010)***	
	Cognitive flexibility		Fregni et al. (2006a)***, Kim et al. (2010)***	
	Verbal fluency/retrieval		Fregni et al. (2006a)***	
	Learning and memory (intermediate-/long-term storage)			
	Verbal learning + memory	Thiel et al. (2013)***	Kim et al. (2010)***	
	(Visual) Associative learning + memory		Kim et al. (2010)***	
	Psychomotor speed			
	Psychomotor speed/processing speed		Kim et al. (2010)***	
Other Organic Disease	Attention			
	Alertness/simple reaction	Smith et al. (2007)*, Fregni et al. (2006b)***, Koren et al. (2001)*		
	Selective/focussed attention		Fregni et al. (2006b)***, Kang et al. (2009)***	
	Executive functions/working memory			
	Working memory (short-term storage/manipulation/monitoring)	Fregni et al. (2006b)***	Kang et al. (2009)***	
	Cognitive flexibility	Fregni et al. (2006b)***	Kang et al. (2009)***	
	Psychomotor speed			
	Psychomotor speed/processing speed	Koren et al. (2001)*		

*** Low-bias risk, ** Medium-bias risk, * High-bias risk

### Risk of bias

**Fig. 2:**
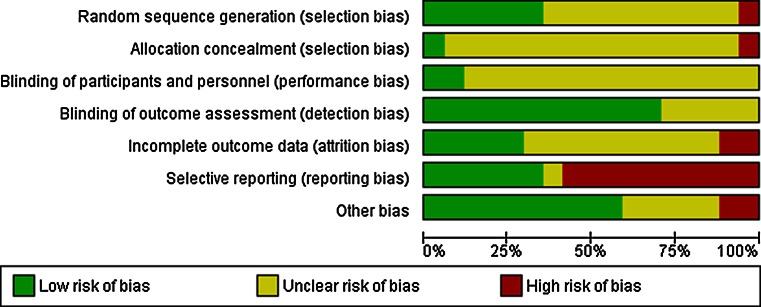
Risk of bias per domain for the included studies

Studies were also marked with asterisks according to the strength of their methodology (*** = Low-bias risk, ** = Medium-bias risk, and * = High-bias risk). Bias assessment was calculated using RevMan 5.1 (Fig. 2). Categories of bias included randomization, allocation concealment, blinding of participants and personnel, blinding of outcome assessment, incomplete outcome data, selective outcome reporting, and others. Studies were rated as high risk, medium risk, and low risk depending on the highest risk level of any individual subcategory. Selective reporting was the most common serious source of bias in the studies (>50 % of included studies, see Fig. 2).

## Discussion

This systematic review overall supports the general safety of rTMS and lack of harm to cognitive functioning (Anderson et al. 2006; Guse et al. 2010). Only two studies reported a significant deterioration (Fitzgerald et al. 2005; Hansen et al. 2011) in the cognitive domain of verbal fluency and retrieval. However, one study (Fitzgerald et al. 2009) found a significant improvement in verbal fluency after rTMS and three studies (Fregni et al. 2006b; Little et al. 2000; Speer et al. 2001) demonstrated no significant effect. The inconsistency of these results may reflect the variation in the methods and outcomes used to assess the impact of 1 Hz rTMS on cognitive functioning. In particular, over 40 different tests assessing various cognitive domains were used with large timing variations as to when subjects were assessed at follow-up. Furthermore, the risk of bias hinders the validity of results, with selective outcome reporting being of particular concern. Incomplete and inadequate outcome reporting is potentially a consequence of cognitive assessment being a secondary outcome in the majority of studies.

### Technical factors

#### Type of coil and sham technique

Several coil factors may influence the effects of rTMS: the type of coil used, sham technique, and positioning during the trial (Lang et al. 2006). Two types of coil were used, the figure of eight coil and the circular coil. Circular coils produce a diffuse magnetic field over a large area and due to this lack of focality they are less used (Wassermann et al. 2008). The adequacy of the sham conditions can be challenged in some studies: in several, it involved placing the coil at a 45° angle away from the skull, which has been shown to still modulate cortical activity (Lisanby et al. 2001; Loo et al. 2000). For example, Lisanby et al. demonstrated that the tilt-induced voltage levels only 24 % below those of active stimulation (Lisanby et al. 2001). Furthermore, one study did not provide detail regarding the degree of tilt (McIntosh et al. 2004). Tilt may also affect blinding due to sensory differences to motor threshold (MT) assessments prior to treatment (Fregni et al. 2006c). Similarly, in those studies using a sham coil, different scalp sensations could unblind patients not naïve to rTMS (Fregni et al. 2006b). The purpose of sham conditions remains to find a protocol that mimics the cutaneous feelings of rTMS (Arana et al. 2008), but thus far, an adequate protocol has yet to be found (Rossi et al. 2001, 2009).

#### Coil positioning

Coil positioning varied between trials. One method involves locating the desired area of stimulation based on its spatial relationship to a functionally determined area, such as the motor cortex (Sparing et al. 2008). For example, to place the coil on the left DLPFC, five centimetres are measured anteriorly in a parasagittal plane from the location where the MT is determined (Nahas et al. 2007). However, the individual-level precision of such generic localisation can be challenged. For example, Herwig et al. found that the 5 cm standard method of locating the DLPFC was accurate in only 7 of 22 included participants (Herwig et al. 2001). Another method of coil positioning uses the electroencephalographic (EEG) international 10–20 system (Jasper 1958), relying on the location of cranial landmarks (e.g., nasion and preauricular points) with the coil placed at set distances from these landmarks (Nahas et al. 2007): once again, this technique is hindered by inter-individual morphological variation (Rusjan et al. 2010).

To improve the precision of stimulation, an increasing number of studies use neuronavigational methods to guide coil positioning e.g. (Herwig et al. 2003; Luber et al. 2008; Smith et al. 2007). Optical frameless stereotaxic systems incorporate imaging data and enable the coil to be positioned via three-dimensional navigation (Lefaucheur 2010; Sparing et al. 2008). Imaging data can be obtained on an individual basis using magnetic resonance imaging (MRI), functional MRI (fMRI) or positron emission tomography (PET), or utilising probabilistic imaging data from large data sets (Lefaucheur 2010; Sparing et al. 2008). However, despite the prima facie improvement in accuracy offered by neuronavigation, a large randomized controlled trial failed to demonstrate superior efficacy of fMRI-guided rTMS was not superior to conventionally applied rTMS or sham stimulation among patients with treatment-resistant auditory verbal hallucinations (AVH) (Slotema et al. 2011).

### Stimulation protocol

Stimulus intensity is determined in relation to the MT (Nahas et al. 2007), and in the majority of studies, it ranged between 80 and 110 % of the MT. Such variation may affect the consistency of results; work on corticospinal excitability has shown, for example, that 115 % rTMS led to a reduction in motor evoked potentials (MEPs), whereas when given at 85 % of the MT, it did not (Fitzgerald et al. 2002). The choice of correct MT threshold has been relatively underexplored in clinical rTMS protocols despite evidence to suggest that both age and medication exhibit significant effects on the MT.

rTMS is known to be less effective among older participants (Figiel et al. 1998; Su et al. 2005). One explanation for this is the increased distance between scalp and cortex among older adults due to age-related cerebral atrophy, with the strength of the magnetic field drops exponentially with distance from the coil (Wassermann et al. 2008). To compensate for cerebral atrophy, the intensity of rTMS can be adjusted considering that the rate of atrophy is not symmetrical across cortical areas (Stokes et al. 2005, 2007). In a sample of depressed patients with an age range of 55–75 years with MT adjusted for distance between the scalp and the cortex, the intensity of rTMS ranged from 103 to 141 % of the MT. Out of 18 patients, four achieved remission and a further five were partial responders (Nahas et al. 2004). These results suggest that correcting for age-related atrophic changes may improve rTMS outcomes in older patients.

Medication may also affect the required rTMS stimulus intensity, and, for example, both the antidepressants citalopram (Minelli et al. 2010; Robol et al. 2004) and clomipramine (Minelli et al. 2010) have been shown to increase the necessary MT. AEDs, such as lamotrigine and phenytoin, increase the MT due to their blocking action on voltage-sensitive sodium channels (Paulus et al. 2008; Wassermann et al. 2008). Long-term use of benzodiazepines also significantly increases the MT (Palmieri et al. 1999). The majority of participants in the included studies were on psychotropic medication that may have affected cognitive functioning and cortical excitability, although this was inconsistently reported in trials, and few evaluated this as a confounder. Anti-epileptic drugs (AEDs), antipsychotics, antidepressants, and benzodiazepines all have potential adverse cognitive side effects (Drane and Meador 2002; Elie et al. 2009; Hori et al. 2012; Schachter 2007). Evaluation of drug effects can be difficult due to the considerable variation between the various drugs, individual susceptibility to side effects, and fluid changes in cognition from functional aspects of the illness itself. For any meaningful comparison to be made between studies, full reporting of study design must be undertaken.

If comparisons are to be made between studies, reporting of study design is of paramount importance. Stimulation parameters, including the number of pulses, the train duration/inter-train interval, and the number of sessions varied considerably or were not consistently recorded: 16 out of the 20 studies did not adequately report these parameters.

Age is also a confounding factor, and similarly underexplored, in relation to neurocognitive testing. In addition to age-related changes to the MT, age-related decline of performance on various cognitive tasks is well documented, e.g., (Brickman et al. 2007; Wielgos et al. 1999). The age range of participants in the included studies was 16–79 and a wide age range was used in each individual study. However, most studies did not report the mean age of their participants making it impossible to draw any conclusions.

## Conclusion

In summary, no definite conclusions can be drawn at this time, regarding the effects of 1 Hz rTMS on cognitive functioning. Calling for more research is futile if that research can produce no meaningful conclusions: to date the lack of unambiguous findings is not due solely to a lack of research—though the field remains underexplored—but is far more hindered by methodological issues. Nevertheless, there are lessons to be learned regarding protocols for rTMS use, confounding factors in studies, and a theory of pre-conditioning and post-conditioning that could greatly improve the quality and applicability of rTMS in mood disorders, psychotic illness, stroke, epilepsy, and other disorders.

There are several clear areas that future research in this field will need to address. The obvious area of need is standardisation—or at least adequate reporting—across several domains: technical; rTMS protocols; and neurocognitive outcome measures. This is true of the broader neuromodulatory field, not limited to cognitive effects. Individual trials are unlikely to be sufficiently powered to elucidate all of these factors, but if they are at least appropriately reported, then bigger data set analysis of these and demographic factors will allow valid cross-comparison and meta-analytic analysis of future work.

The figure of eight coil has largely superseded the circular coil, and it is unlikely much future work will be undertaken with the latter. Blinding and sham condition paradigms remain problematically inconsistent, but despite the issue of the lack of sensation, sham coils appear a better proposition than coil tilting. With regard to coil siting, whilst we note the negative findings of Sloetma et al., it is our opinion that neuronavigation is an inherently superior paradigm as its functional approach addresses individual variation. However, further studies are warranted if this argument is to be proved or disproved.

Standardised cognitive batteries would hugely facilitate across-study comparisons and the replication of studies. The MATRICs initiative in schizophrenia studies is a worthy reference point in this regard. As with MATRICS, good test–retest reliability, practicality of test usage, relationship to functional outcome, response to pharmacological adjuncts, and use as a repeated measure should be characteristics of the included tests (Green et al. 2004). Such protocol consistency is, especially, important given that any protocol discrepancies are compounded by confounding factors intrinsic to the populations studied: in particular, age-related changes in cognition and concomitant pharmacotherapy.

Finally, it may be the case that pre-conditioning and post-conditioning of the brain are necessary to take full advantage of the positive effects of rTMS. This hypothesis of pre-conditioning the brain is already borne out through studies on the effects of pharmaceuticals in rTMS studies (Fregni et al. 2006a), in which the effects of 1 Hz rTMS depended on the state of cortical excitability at the time of stimulation.

In addition to pharmaceutical pre-conditioning, studies have used transcranial direct current stimulation (tDCS) to modulate the effects of 1 Hz rTMS. Siebner et al. demonstrated that excitatory anodal tDCS caused 1 Hz rTMS to further reduce cortical excitability, whereas inhibitory cathodal tDCS led to an increase in excitability following 1 Hz rTMS (Siebner et al. 2004). Similarly, Iyer et al. found that 6 Hz rTMS enhanced the inhibitory effects of 1 Hz rTMS (Iyer et al. 2003). The same pattern of results was also obtained by Lang et al., in which the direction of 5 Hz rTMS was determined by preceding tDCS conditioning of motor cortex excitability (Lang et al. 2004). These findings have potentially important implications for the included studies with a cross-over design, e.g., (Fitzgerald et al. 2009; Little et al. 2000). Since some of these studies crossed-over patients from low-frequency rTMS to high-frequency rTMS and vice versa, it is possible that carry-over effects confounded the results, but also that cross-over studies may compound positive effects.

A further point in this regard is that there is reasonably good basic neuroscience data that rTMS can enhance neuronal plasticity—the mechanism, indeed, that would underlie any putative cognitive enhancement. However, with this in mind, there is an obvious dearth of utilising parallel cognitive remediation during the trial rTMS period.

rTMS has been in existence since the 1980s. Despite this, and the ongoing interest in its potential clinical roles, it remains incompletely understood. There are data to support its utility in several neuropsychiatric disorders and, somewhat more speculatively, to enhance cognition. At this time, it remains unclear how much the somewhat ambivalent data represent the technique’s fundamental limitations, and how much the numerous confounders are clouding any underlying improvement. If practical aspects mean that smaller study size remain the norm, this should at least be done within the context of standardised reporting that will allow work to fit within bigger subsequent data sets.
