# Correction to: A systematic review of the effects of low-frequency repetitive transcranial magnetic stimulation on cognition

**DOI:** 10.1007/s00702-019-02027-7

**Published:** 2019-06-21

**Authors:** Claudia Lage, Katherine Wiles, Sukhwinder S. Shergill, Derek K. Tracy

**Affiliations:** 1 Cognition, Schizophrenia and Imaging Laboratory, The Institute of Psychiatry, Psychology and Neuroscience, King’s College London, London, UK; 2 King’s College London School of Medicine, London, UK; 3 South London and Maudsley NHS Foundation Trust, London, UK; 4 Oxleas NHS Foundation Trust, London, UK

## Correction to: J Neural Transm (2016) 123:1479–1490 10.1007/s00702-016-1592-8

The original version of this article unfortunately contained a mistake. The author would like to include the below acknowledgement section.

